# Supplementary material for: NextVir: Enabling classification of tumor-causing viruses with genomic foundation models
Source: PLoS Comput Biol. 2025 Aug 21;21(8):e1013360. doi: 10.1371/journal.pcbi.1013360 (PMC12396758; doi:10.1371/journal.pcbi.1013360)
Supplement: S1 Appendix — (PDF) [file pcbi.1013360.s001.pdf]

Appendices

A. Choice of the LoRA rank

The results presented thus far are obtained by using the LoRA rank of 4 due to the diminished gains and longer training times when higher values are used. While it is computationally too expensive to run all experiments at a higher rank, we obtained the results for NextVir-D at higher ranks and present them in Table S1.

Table S1. Performance as LoRA rank is varied.

LoRA rank	4	8	16	32
	Accuracy (%)			
Overall top-1	94.69	94.45	95.45	96.02
Human	91.81	91.40	93.07	94.12
HBV	99.74	99.70	99.76	99.74
HPV	95.53	95.29	96.04	96.34
HCV	95.68	96.12	96.23	96.40
EBV	95.13	94.99	95.66	95.02
HTLV	98.18	98.67	98.10	99.24
HHV-8	62.33	65.58	64.65	67.91
MCV	99.05	99.40	99.41	98.82

B. Single species binary detection

In this experiment, we train seven separate NextVir-D classifiers, one corresponding to each oncoviral class. For a given viral class i, the corresponding network is trained using a training set consisting of all the viral reads of class i and an equal number of human reads selected at random from the original dataset. As can be seen from Table S2, each NextVir classifier is highly accurate, with all of them exhibiting accuracies greater than 97%. This is especially noteworthy for the HHV-8 class, which proved the most difficult for NextVir to classify in the multiclass setting, as evidenced by the results presented in Results. The high level of accuracy attained by these binary classifiers can be thought of as empirical ceilings for the per-class accuracy that can be achieved by NextVir in the multiclass setting. Note that the training set for the HHV-8 classifier is rather small (< 4000 reads), so the high accuracy attained by the corresponding classifier may simply be a consequence of memorization, i.e., overfitting.

C. Context-supported dataset

The context-supported classification experiments are motivated by the desire to understand how well the proposed methods learn patterns from the sequencing reads *in silico*, as this in turn may inform us of the utility of these methods in experimental settings. This is particularly important as the deep learning frameworks in genomics keep using increasingly larger models; the large number of parameters renders these models prone to overfitting. The task of context-supported classification can be viewed as a way of assessing a model's ability to learn patterns inherent to various classes.

As described in Context-supported classification, we aim to emulate experimental artifacts that may occur during NGS analysis of a tumor sample. Specifically, a key step in the sequencing pipeline is the amplification of DNA in the sample using the Polymerase Chain Reaction (PCR). This is accomplished through the use of primers which should ideally bind to the ends of each DNA fragment in order to enable replication of the entire fragment. In cases where the primer binds unevenly across fragments from certain regions of the underlying genome, the genome may be covered unevenly by the amplified fragments. In the extreme case, only a section of the genome may be covered by the amplified fragments, and consequently sequencing reads, while the coverage in other regions of the genome is reduced/minimal.

We emulate this extreme scenario by dividing the dataset into disjoint sets such that the reads in any given subset do not overlap with the reads from any other subset. This is achieved by first using BWA-MEM [2] to align all sequencing reads in the dataset to the genomes used to generate the reads; the references include the viral genomes from iCAV database and the primary assemblies from the GRCh38.p14 reference genome. The read alignment assigns each read to a specific position on the genome that the read originates from. This is depicted in Fig. S1 for a single reference genome. With this information, we allocate the reads for each class to the training, validation and test sets in the ratio 8:1:1, such that these sets are disjoint.

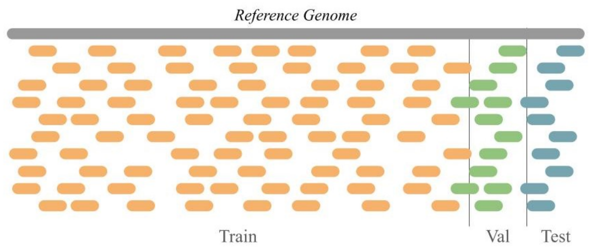

Fig. S1: Visualization of context-supported dataset construction for a single reference genome. The small line segments denote sequencing reads, yellow reads belong to the training set, green to the validation set and blue to the test set.

Table S2. Subsampled single class performance.

Viral target	Accuracy (%)	AUCROC	True positive rate	False positive rate
HBV	99.99	1.000	1.000	4.22×10^{-5}
HPV	98.25	0.999	0.983	0.0177
HCV	98.95	0.999	0.993	0.0136
EBV	96.89	0.995	0.982	0.0445
HTLV	98.96	0.998	0.998	0.0190
HHV-8	93.72	0.980	0.921	0.0465
MCV	97.93	0.999	0.976	0.0178

D. Optimization

As mentioned in Network optimization, two separate grid searches are performed in order to select and tune the hyperparameters of NextVir. The hyperparameters chosen this way are used in all subsequent experiments. NextVir-D is selected for tuning based on its pretrained performance. In practice, we find that the schedule-free optimizer does an excellent job of converging to satisfactory performance for all models.

First, we compare the performance across different learning rates. We focus at the setting where the initial learning rate for DNABERT-S is 1/10th of the adapter's. This setting is chosen for its speed/efficiency; validation loss is minimized in only 2 epochs. Since we are studying the initial learning rates, information from shorter runs should sufficiently inform the final framework. For the first grid search, there is no weight decay or regularization tuning. The best result can be seen in Table S3, with learning rates set to 10^{-3} minimizing validation loss.

Table S3. Grid search 1 over 4 epochs. LR refers to learning rate

LR ratio	Initial LR	Val. accuracy (%)	Val. loss
1	5×10^{-4}	98.31	4.96×10^{-4}
1	1×10^{-3}	**98.27**	$\mathbf{4.48 \times 10^{-4}}$
1	5×10^{-3}	64.45	4.86×10^{-3}
1/10	5×10^{-4}	97.68	4.82×10^{-4}
1/10	1×10^{-3}	98.06	4.70×10^{-4}
1/10	5×10^{-3}	98.32	4.90×10^{-4}

In the second grid search, we investigate how weight decay (λ) and regularization through β_1 may improve longer training runs. All models are trained for 15 epochs. As can be seen in Table S4, validation loss is minimized using a weight decay value of 0.005 and β_1 set to 0.85. The tuning for hyperparameters is based on the guidelines provided in the schedule-free repository, linked in **??**.

Table S4. Grid search 2 over 15 epochs. Showing validation loss.

β_1	Weight decay (λ)		
	0.001	0.005	0.01
0.85	0.0006199	**0.0006022**	0.0006809
0.90	0.000714	0.0007733	0.000773
0.95	0.0008578	0.0008327	0.000817

E. Simulated viral discovery

Here, we study a NextVir-D model trained on six out of 7 viruses in the binary setting, and tested on a dataset comprised of an equal number of sequencing reads from the human genome and the genomes from the held-out viral class. The desired outcome for a multi-class model in this setting is that by virtue of learning "good" embeddings for the negative (human) class, the model is able to correctly identify reads from a hitherto unseen virus.

Table S5. NextVir-D performance detecting an unknown viral class

Unknown viral target	True positive rate	AUCROC
HBV	0.427	0.933
HPV	0.107	0.849
HCV	0.798	0.987
EBV	0.435	0.912
HTLV	0.472	0.948
HHV-8	0.758	0.982
MCV	0.231	0.874

AUCROC is computed based on the classification of reads in a test set constructed with the sub-sampling process described in Appendix B, i.e., containing all the viral test samples from the target class and subsampling the human test samples so that there is an even class distribution. The true positive rate (TPR) only counts viral reads from the unseen class that are predicted to be of viral origin with a probability greater than 0.5. On the other hand, the AUCROC rewards models which attribute higher probabilites to reads from the unseen viral class over other reads (from the negative class). As can be seen from Table S5, the AUCROC is consistently quite high. This indicates that the model is able to generalize from viral reads to unseen viral classes, in that it consistently deems reads from the latter class to be more likely to be of viral origin than the human reads. However, the model is not confident in this determination, as can be observed from the low TPR values on some classes. Coupled with the high AUCROC, this suggests that the NextVir-D model is well-calibrated; it is most confident at recognizing viral reads that it saw during training, while also ranking reads from unseen viruses over human reads in terms of their probability of viral origin.

In addition to this, the high values of TPR for HCV and HHV-8 indicates that from the model's perspective, there is some overlap in the latent representation of these classes with those belonging to the remaining corpus of DNA (including each other). Moreover, it appears that a lot of this "information overlap" arises from the HPV class; not only is this the second-most abundant class of

Table S6. NextVir-D classification performance on uniformly sampled test data.

Class	Accuracy (%)
Overall Top-1	94.98
Human	92.53
HBV	99.77
HPV	95.29
HCV	95.96
EBV	95.63
HTLV	97.52
HHV-8	58.42
MCV	100

reads, but withholding reads from this class during training results in the lowest TPR and AUCROC. Further study is required to fully understand how the foundational models are extracting underlying information from viral families during training in order to identify reads from unseen viral families.

F. Results on uniformly sampled data.

For the binary setting involving new uniformly sampled set, NextVir-D achieved an accuracy of 98.18 and an AUCROC of 0.998. Additionally, Table S6 indicates little change in multiclass performance as well. This is almost exactly the same setting originally studied, with the only difference being that the uniform sampling is done per-class, ensuring exactly the same class imbalance across the splits. In the previous experimental settings, the whole set is uniformly sampled to make the train/test/val split. These results demonstrate that there is little difference between uniformly sampling our entire read dataset and uniformly sampling per-class, which is expected.

G. Difficulty detecting HHV-8

Across our various experiments involving multi-species classification, HHV-8 consistently emerges as the most challenging viral class to identify. As we previously hypothesized, this might be caused by its low coverage relative to other viruses and sequence similarities with other viral genomes. In this section, we provide additional metrics to further investigate performance limitations specific to HHV-8.

Table S7. Additional classification metrics for the HHV-8 class in multi-species identification

Model	F1-score	Precision	Recall
NextVir-D	0.281	0.184	0.595
NextVir-N	0.272	0.177	0.591
NextVir-H	0.193	0.116	0.586

Table S7 reports F1-score, precision, and recall for the HHV-8 class across the NextVir models. For context, the F1 score for a randomly-guessing baseline across 8 classes would be 0.125. While all models achieve relatively strong recall scores (also reported as per-class accuracy elsewhere), the low precision values suggest that other reads are frequently misclassified as HHV-8. This indicates a knock-on effect of HHV-8's low coverage: the model's limited exposure to HHV-8 reads results in a poor class representation, which in turn leads to misclassification of reads from other classes as HHV-8. In other words, reads with sequence similarity to HHV-8 are prone to being false positive. This exposes a limitation of the current loss-weighted approach to handling class imbalance. Potential future work may investigate alternative strategies, such as synthetic data augmentation to increase HHV-8 coverage (albeit requiring caution as that may cause experimental artifacts), an alternative loss-weighting mechanism, or approaches that incorporate alignment-based post-processing to help reduce false positives in low-coverage classes such as HHV-8.

H. Detecting HPV in experimental data

In this section, we explore the potential of NextVir models to classify oncoviral reads in real-world experimental data. This setting is the primary intended use case of the NextVir framework, yet its experimental verification presents significant challenges. Most notably, experimental data lacks read-level ground truth annotations, i.e., there is no definitive labeling that would indicate the true genomic origin of the reads. This limitation renders rigorous evaluation of read-based classification methods like NextVir inherently difficult. To circumvent this, our main results are based on semi-experimental data comprising 150bp Illumina-style reads generated using the ART simulator. This strategy allows us to closely emulate the complexity of real sequencing data, while still creating a dataset with read-level labels derived from known reference genomes. Compared to alternative synthetic data generation approaches, such as the popular random contig sampling for longer sequences [4, 5], ART offers a key advantage by introducing realistic sequencing errors and biases characteristic of real-world Illumina platforms.

To demonstrate the feasibility of applying NextVir to real sequencing data, we evaluate its performance on cervical tumor samples obtained from [1]. In that study, each sample underwent both deep sequencing and PCR-based analysis using 150bp paired-end reads to detect the presence of HPV DNA. Each sample is annotated with the outcomes of predictions by both methods. For this analysis, we selected eight samples – four that are labeled positive by both sequencing and PCR, and four that are labeled positive by deep sequencing but negative by PCR. From each sample, we randomly subsample 1 million reads to reduce the inference time while maintaining statistical significance of the results.

NextVir models are applied to the 8 million experimental reads using the same training configuration as in Appendix B, with the goal of detecting HPV-positive reads. NextVir-D predicted HPV in 4.69% of reads in samples identified as HPV-positive by both methods, and in 3.86% of the reads from samples where the two methods disagreed. NextVir-N produces similar results, detecting HPV in 5.85% and 4.14% of reads in the respective groups. NextVir-H yielded lower detection rates – 1.51% and 1.22%, respectively – consistent with its comparatively lower performance in prior analyses. Notably, estimates from [1] suggest that approximately 4% of reads in these datasets are of HPV in origin. The predictions from NextVir-D and NextVir-N closely track these estimates, offering preliminary evidence that these models may generalize effectively to real sequencing data, despite being trained exclusively on simulated reads.

Without definitive knowledge of which reads originate from the host or from HPV, more granular evaluation remains infeasible. One notable observation is that NextVir-H consistently detects fewer HPV reads than the other models. We hypothesize that HyenaDNA's architecture may be less effective at classifying shorter reads, especially when they deviate in length from the reads used in pretraining or fine-tuning. This issue appears relevant in experimental data, where sequencing often terminates early, sometimes well before the full 150 bases, due to uncertainty in base calling. A promising direction for future work may involve augmenting HyenaDNA-based models with training data that includes a range of read lengths to improve robustness in such settings.

I. Additional benchmarks versus binary viral detection methods

I.1. Detecting oncoviruses in gold-standard datasets

In the absence of publicly available gold-standard dataset containing labeled oncoviral reads, we constructed a benchmark using the CAMI II Toy Human Microbiome dataset as a substitute for the human (non-viral) background in our experiments. This dataset is a widely used resource for evaluating metagenomic analysis tools, comprising 49 simulated samples drawn from five human body sites. Generated from 1,680 microbial genomes, the dataset includes both Illumina short-read and PacBio long-read data, allowing for comprehensive performance evaluation across sequencing platforms. Designed to reflect realistic microbial community compositions while preserving read-level ground truth, CAMI II enables precise validation of classification, assembly, binning, and taxonomic profiling tools [3].

Although the CAMI II dataset is well-suited for benchmark metagenomic binning and taxonomic profiling, its incomplete labeling of host-virus relations and overall complexity makes it unsuitable for direct evaluation of oncoviral detection using our existing binary models [3]. To address this, we recreate our original train, validation, and test datasets using the same procedures as before, but substitute randomly sampled 150 bp reads from the CAMI toy human microbiome dataset as the negative (non-oncoviral) class. As a result, we emulate a more challenging yet controlled binary classification task, reflecting real-world conditions while working with accurate labels.

Table S8. Performance of binary methods with CAMI II Toy Human Microbiome as the negative class

Method	Accuracy	AUCROC
NextVir-D	94.68	0.989
NextVir-N	98.59	0.999
NextVir-H	97.81	0.998
DVF	99.03	0.999
XVir	98.00	0.998
Virtifier	95.30	0.991

As shown in Table S8, NextVir maintains strong performance even when evaluated on this more complex dataset comprising a mixture of human microbiome reads. These results provide additional support for potential suitability of NextVir for use in oncogenetics pipelines. Specifically, the framework demonstrates the ability to reliably distinguish oncoviral reads from a realistic background of microbial and host DNA. In practice, this suggests that any reads originating from cancer-associated bacterial species are likely to be classified as non-oncoviral by NextVir, making them available for downstream analysis using bacterial-specific classifiers.

I.2. Effects of sequence length on oncoviral detection

While the 150bp reads used throughout most of this study closely mimic Illumina MiSeq data, it is important to assess whether NextVir can generalize to data from other sequencing platforms or experimental conditions. To this end, we evaluate the model performance on longer sequences by simulating 250bp reads (typical of MiSeq), as well as 500bp and 1kbp contigs. The 250bp reads are generated using ART with the error profile setting of Illumina MiSeq v3 reads. For contigs, we follow the procedure described in [4, 5], sampling contiguous fragments of the specified length from the reference genome without replacement.

As shown in Table S9, NextVir achieves consistently strong performance across all tested sequence lengths, suggesting its potential as a new benchmark for oncoviral detection on longer DNA fragments. Both NextVir-D and NextVir-H outperform existing state-of-the-art methods on 1 kbp contigs, with near-perfect AUC-ROC scores observed across all sequence lengths. Due to hardware constraints, evaluating the Nucleotide Transformer on longer sequences appears challenging. Its large model size and memory footprint make it infeasible to train with longer inputs under our current setup without significantly reducing batch size. This, in turn, would necessitate a new hyperparameter search, which is computationally intensive. Given the already strong performance of NextVir-D and NextVir-H, we concluded that these results are sufficient to demonstrate the framework's ability to generalize across varying sequence lengths.

I.3. Computational requirements

In this section, we assess the computational requirements of training and running current state-of-the-art (SoTA) methods for binary viral detection, alongside the NextVir models. This is an essential consideration for the broader adoption of deep learning frameworks in computational biology, where many users may lack access to high-end GPU infrastructure. The shared Linux server described in Section Hardware and software serves as a reasonable approximation of the hardware likely available to downstream researchers, striking a practical balance between capability and accessibility. Note that it is also important to consider the software ecosystems that is required/suitable for running these methods. GPU acceleration is enabled for models implemented in PyTorch (e.g., XVir), but this is not feasible for older frameworks. Specifically, DeepVirFinder and Virtifier rely on legacy backends (Theano and TensorFlow v1, respectively) which are no longer actively maintained and lack support for modern GPU hardware. As a result, these methods are run exclusively on CPU, limiting their efficiency and complicating direct runtime comparisons.

Table S10 reports the average inference latency, training time, and training memory consumption for the methods studied in this work. These are measured using the dataset described in Appendix I.1, matching the original dataset in size and sequence length. Training time is measured using the real-world clock time, and includes preprocessing and validation. To calculate inference

Table S9. Performance of binary methods at different sequence lengths

Method	250bp Reads		500bp Contigs		1kbp Contigs	
	Accuracy (%)	AUCROC Score	Accuracy (%)	AUCROC Score	Accuracy (%)	AUCROC Score
NextVir-D	97.50	0.997	99.73	1.000	99.92	1.000
NextVir-N	99.56	1.000	N/A	N/A	N/A	N/A
NextVir-H	99.06	0.999	99.87	1.000	99.93	1.000
DeepVirFinder	99.41	1.000	99.85	1.000	99.78	1.000
XVir	98.90	0.999	99.60	1.000	99.60	1.000
Virtifier	96.20	0.994	98.90	0.999	99.20	0.999

Table S10. Computational efficiency of binary viral detection methods

Method	Inference latency (s)	Training time (s)	Training memory usage (MB)
NextVir-D	3.81E-03	20525	8340
NextVir-N	8.51E-03	58600	7870
NextVir-H	3.72E-03	34811	6860
DeepVirFinder	0.565	56498	8790
XVir	8.00E-04	29422	4062
Virtifier	9.99E-04	21972	86646

latency, the ”prediction” scripts for each method are timed, and the total time to predict on every read in the test set is divided by the total number of reads. Memory usage is assessed using the Linux ”ps” command after training had stabilized (typically 20-30 minutes into a training run). As most methods aggressively allocate memory (with virtual memory usage approaching 400 GB), we report the resident set size (RSS) as a more realistic estimate of actual memory consumption.

These results highlight significant differences in computational efficiency across methods. Despite its strong classification accuracy, DeepVirFinder (DVF) suffers from slow inference and prolonged training time. This is likely due to its reliance on Theano, a framework that was already deprecated at the time of DVF's publication. In contrast, Virtifier benefits from slightly more modern backend support and is reasonably efficient, with the primary limitation being its high memory consumption during training. The NextVir models offer a balance of computational efficiency and predictive accuracy. In particular, NextVir-D achieves low inference latency, moderate memory usage, and rapid training time, making it especially suitable for deployment in practical settings where computational resources may be constrained.

References

1. Laila Sara Arroyo Mühr, Camilla Lagheden, Jiayao Lei, Carina Eklund, Sara Nordqvist Kleppe, Pär Sparén, Karin Sundström, and Joakim Dillner. Deep sequencing detects human papillomavirus (hpv) in cervical cancers negative for hpv by pcr. *British journal of cancer*, 123(12):1790–1795, 2020.
2. Heng Li and Richard Durbin. Fast and accurate short read alignment with burrows–wheeler transform. *bioinformatics*, 25(14):1754–1760, 2009.
3. Fernando Meyer, Adrian Fritz, Zhi-Luo Deng, David Koslicki, Till Robin Lesker, Alexey Gurevich, Gary Robertson, Mohammed Alser, Dmitry Antipov, Francesco Beghini, Denis Bertrand, Jaqueline J. Brito, C. Titus Brown, Jan Buchmann, Aydin Buluç, Bo Chen, Rayan Chikhi, Philip T. L. C. Clausen, Alexandru Cristian, Piotr Wojciech Dabrowski, Aaron E. Darling, Rob Egan, Eleazar Eskin, Evangelos Georganas, Eugene Goltsman, Melissa A. Gray, Lars Hestbjerg Hansen, Steven Hofmeyr, Pingqin Huang, Luiz Irber, Huijue Jia, Tue Sparholt Jørgensen, Silas D. Kieser, Terje Klemetsen, Axel Kola, Mikhail Kolmogorov, Anton Korobeynikov, Jason Kwan, Nathan LaPierre, Claire Lemaitre, Chenhao Li, Antoine Limasset, Fabio Malcher-Miranda, Serghei Mangul, Vanessa R. Marcelino, Camille Marchet, Pierre Marijon, Dmitry Meleshko, Daniel R. Mende, Alessio Milanese, Niranjan Nagarajan, Jakob Nissen, Sergey Nurk, Leonid Oliker, Lucas Paoli, Pierre Peterlongo, Vitor C. Piro, Jacob S. Porter, Simon Rasmussen, Evan R. Rees, Knut Reinert, Bernhard Renard, Espen Mikal Robertsen, Gail L. Rosen, Hans-Joachim Ruscheweyh, Varuni Sarwal, Nicola Segata, Enrico Seiler, Lizhen Shi, Fengzhu Sun, Shinichi Sunagawa, Søren Johannes Sørensen, Ashleigh Thomas, Chengxuan Tong, Mirko Trajkovski, Julien Tremblay, Gherman Uritskiy, Riccardo Vicedomini, Zhengyang Wang, Ziye Wang, Zhong Wang, Andrew Warren, Nils Peder Willassen, Katherine Yelick, Ronghui You, Georg Zeller, Zhengqiao Zhao, Shanfeng Zhu, Jie Zhu, Ruben Garrido-Oter, Petra Gastmeier, Stephane Hacquard, Susanne Häußler, Ariane Khaledi, Friederike Maechler, Fantin Mesny, Simona Radutoiu, Paul Schulze-Lefert, Nathiana Smit, Till Strowig, Andreas Bremges, Alexander Sczyrba, and Alice Carolyn McHardy. Critical Assessment of Metagenome Interpretation: The second round of challenges. *Nature Methods*, 19(4):429–440, April 2022.
4. Jie Ren, Nathan A Ahlgren, Yang Young Lu, Jed A Fuhrman, and Fengzhu Sun. Virfinder: a novel k-mer based tool for identifying viral sequences from assembled metagenomic data. *Microbiome*, 5(1):1–20, 2017.
5. Jie Ren, Kai Song, Chao Deng, Nathan A Ahlgren, Jed A Fuhrman, Yi Li, Xiaohui Xie, Ryan Poplin, and Fengzhu Sun. Identifying viruses from metagenomic data using deep learning. *Quantitative Biology*, 8:64–77, 2020.
