# Supplementary material for: NextVir: Enabling classification of tumor-causing viruses with genomic foundation models
Source: PLoS Comput Biol. 2025 Aug 21;21(8):e1013360. doi: 10.1371/journal.pcbi.1013360 (PMC12396758; doi:10.1371/journal.pcbi.1013360)
Supplement: Attachment — Submitted filename: NextVir_revision_response.pdf [file pcbi.1013360.s002.pdf]

Response to reviews

Reviewer #1:

The manuscript by Robertson et al. presented a multi-class viral classification tool that adapts three genomic foundation models to detect oncoviruses from sequencing reads. The results showed that foundational models can be fine-tuned to perform viral classification at high accuracy, with performance comparable to well-established binary viral classifiers. However, several major issues should be carefully addressed before it can be published as a rigorous research paper, listed below.

1. NextVir is designed to identify oncoviruses from cancer cells. However, there are also diverse bacteria (Tekle et al., 2023) that are known or suspected to cause cancer. In addition, samples can be easily contaminated by environmental bacteria during sampling, processing, DNA extraction, library preparation, and sequencing. Thus, these scenarios should be considered during the design of NextVir or other similar tools.

We thank the reviewer for the important observation regarding the presence of bacterial sequences in cancer samples, whether due to contamination or from potentially oncogenic bacteria. We agree that sequencing data often include reads of non-human/non-viral origin, and that distinguishing such reads is an important practical consideration. To assess the robustness of our approach in such realistic scenarios, we evaluated the performance of NextVir across varying levels of contamination in the test data. As reported in Figure 2 (and shown below), NextVir maintains stable classification performance even under high levels of bacterial contamination, demonstrating the method's resilience to contaminants. Please recall that NextVir is designed specifically to identify oncoviral reads; all other reads, including those of bacterial origin, are assigned to the negative class (referred to as the "human" class in the originally submitted manuscript). While fine-grained classification within the negative class is beyond the current scope of NextVir, we note that specialized tools such as DeepMicrobes or MetaTransformer can be applied post hoc to further analyze these reads and identify potential bacterial content. We've amended the manuscript to include a Robustness to contamination section which covers this, and new Appendix I.2 provides further arguments for this usage of NextVir.

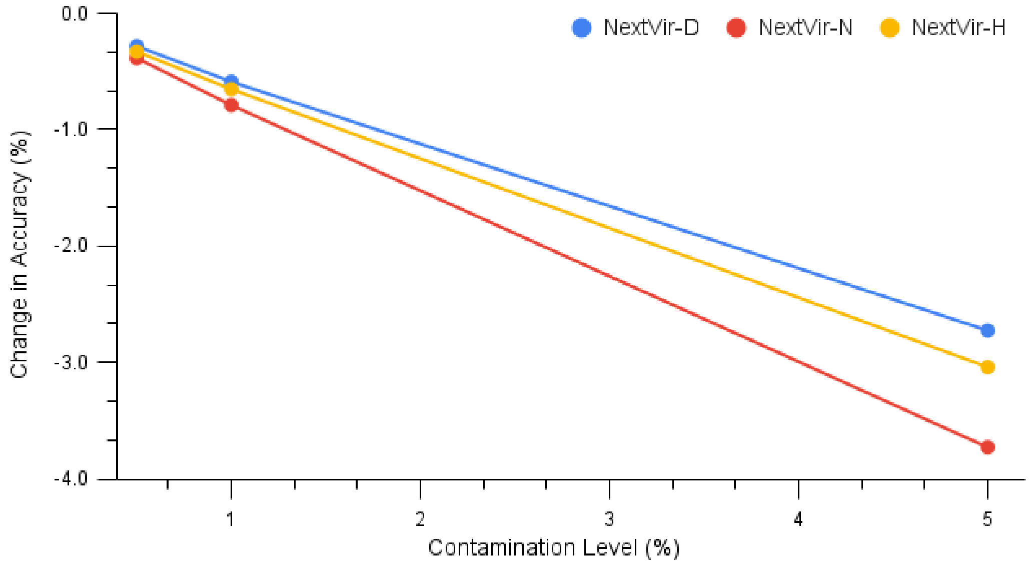

2. The training dataset was semi-experimental, so it may not perfectly emulate actual sequencing data, particularly the diversity, highly variable coverage, and complexity of actual samples. Actually, the accuracy dropped significantly with the increase of mutations, and particularly indels, which are typical for real samples.

We appreciate the reviewer's concern regarding the use of semi-experimental data for training. However, fully experimental datasets that support comprehensive performance benchmarking under controlled yet varied conditions are difficult to obtain. As a compromise, we rely on samples with confirmed viral presence (established through PCR assays or validated deep sequencing pipelines) and evaluate a binary version of NextVir on such experimental cervical cancer samples to assess its practical utility in identifying HPV reads. As shown in Appendix H, the model successfully detects HPV in these real samples despite inherent biological and technical noise, including mutations and potential indels.

3. When benchmarking NextVir with other classifiers, the authors should A) use golden standard real-world metagenomes of human-associated, soil, marine, and other well-studied environments to capture the complexity of metagenomic samples. B) the authors should benchmark them at different read/contig lengths since the reads can be generated from different sequencing platforms, and it's a common practice to detect viruses from assembled contigs, particularly those with long sequence lengths but low sequencing coverage. C) the authors should also compare the computational and memory costs when benchmarking these tools, which is vital to be adopted by the community.

We thank the reviewer for these thoughtful suggestions regarding benchmarking strategies. We address each point below:

A) We agree that benchmarking on real-world metagenomes would add valuable insight. However, to our knowledge, there are currently no gold-standard metagenomic datasets with labeled oncoviral reads across diverse environments that could be used to perform such benchmarking experiments. Nevertheless, following up on the reviewer's suggestion, we evaluated NextVir on the CAMI II Toy Human Microbiome dataset, which includes human reads as well as a complex mixture of reads from microbial sequences commonly found in the human body. To simulate a more realistic background, we replaced the human reference genome reads in our original dataset with reads from this CAMI II dataset. As shown in Appendix I.1, NextVir maintains near–state-of-the-art viral classification accuracy under these conditions, demonstrating its robustness even in the presence of diverse background DNA.

B) We have now included benchmarking of NextVir and other methods across a range of sequencing read lengths. Specifically, we used ART to simulate 250 bp reads and followed the procedure previously utilized by DeepVirFinder to generate 500 bp and 1 kbp contigs. Results reported in Appendix I.2 show that NextVir remains highly accurate across all tested lengths, and even outperforms other methods at longer sequence lengths.

C) Thank you for the suggestion! We have added a comparative analysis of training and inference costs for all evaluated methods in Appendix I.3. Please note that DeepVirFinder and Virtifier are incompatible with AMD GPUs used in our study and thus required CPU-based training. Additionally, these methods depend on legacy backends (Theano and TensorFlow v1, respectively), which limits their compatibility with modern hardware and software environments. In

contrast, NextVir supports GPU acceleration and uses up-to-date frameworks, making it more practical for future deployment and integration.

Reviewer #2:

The authors present a novel method for multi-class oncoviral read detection and classification utilizing next-generation sequencing data. The proposed method, NextVir, builds on several recent genomic foundation models, including DNABERT-S, NucleotideTransformer, and HyenaDNA, by fine-tuning and adapting their read embeddings to achieve accurate classification of reads based on their origin. Notably, NextVir also achieves state-of-the-art performance in binary viral detection tasks. Overall, the proposed framework has a superior performance and can solve not only the task of oncolytic virus read classification, but also various other problems in genomics.

1. In the Methods section, under Input Preprocessing, please provide a more detailed explanation of the padding method used and indicate whether it will have an effect on the final result.

We thank the reviewer for this feedback. Padding is performed by using a special [PAD] token at the ends of shorter sequences in a batch. By generating associated attention masks and applying them to the batched inputs, we can ensure that attention operations only attend to the tokens which pertain to the read and ignore the padding. This allows us to train with sequences of variable lengths, and is a widely used practice with transformers due to the inefficiency of training on a single sequence at a time. We've further elaborated on this operation and provided more detail in the *Input Preprocessing* section to eliminate readers' confusion.

2. The authors propose a NextVir approach based on three foundation models, DNABERT-S, NucleotideTransformer, and HyenaDNA. It is recommended to discuss the advantages and disadvantages of the three models in depth, describing the scenarios and potential limitations of each model.

Thank you for this excellent suggestion. We now dedicate the second paragraph of the new *Discussion* section to comparing the three foundation models used in our work. We hope this will provide readers with the ability to discern which model may be best suited for their use case. In summary, we find DNABERT-S to be the easiest to use while maintaining high-accuracy, but NucleotideTransformer and HyenaDNA may be suitable when higher performance or efficiency is required, respectively.

3. It is recommended that the authors explore the possibility of extending the NextVir approach to other foundational models. This will help to assess the generalizability of NextVir and its potential for application in different genomics tasks.

We appreciate the reviewer's suggestion to explore the generalizability of the NextVir approach to other foundational models. Indeed, a key strength of the proposed framework is its modularity – NextVir is not tied to any specific genomic foundation model (GFM). This is demonstrated by our use of three distinct GFMs (HyenaDNA, DNABERT-S, and NucleotideTransformer), each with substantially different architectures and capacities. The strong and consistent performance of NextVir across these models suggests that the proposed framework is likely to remain effective when paired with even larger or more advanced GFMs. That being said, we acknowledge that evaluating additional GFMs would further reinforce this point; however, such experiments are currently limited by resource constraints, as many of the newest models (e.g., EVO) require extensive memory and high-end GPU infrastructure. In place of

additional GFM comparisons, we investigated other critical aspects of generalizability, such as sequence length variations shown in Appendix I.2.

4. It is recommended that the authors further discuss whether it is possible to further improve the performance of the model under low coverage conditions.

We appreciate the reviewer's interest in the model's performance under low-coverage conditions, which represent a critical challenge in classification tasks on sequencing data. Note that our choice of weighted cross-entropy loss over cross-entropy loss was motivated in part by the low coverage in the sequencing data, and has resulted in improved performance of the proposed framework in low-coverage settings. We also note that accurate prediction in extremely low-coverage classes is particularly challenging in the multi-class setting. In this context, NextVir's ability to classify HHV-8 (the sparsest class in the dataset) with over 50% accuracy (as shown in Table 2) is a reassuring result, especially given the limited number of training examples and competing signals from more prevalent classes. Further discussion of the challenges associated with low-coverage classes, including HHV-8, has been added to Appendix G, which also outlines possible future work to mitigate these challenges.

5. The authors mention that despite the scarcity of MCV reads, its genome length is much shorter than that of HHV-8, which may account for the more stable accuracy of MCV classification. It is recommended that the authors further explore the specific impact of genome length on model performance.

Thank you for this thoughtful suggestion. We would like to clarify that genome length, in itself, does not necessarily impact model performance. Rather, it is sequencing coverage that plays a critical role. For two viral families with a similar number of sequencing reads, the class with shorter genome length will have higher sequencing coverage, which in turn facilitates more accurate classification of the reads from that class. In the case of MCV, its relatively short genome length results in higher coverage than for HHV-8, despite the similar scarcity of reads; this likely contributes to the more stable classification accuracy observed for MCV. As discussed in Appendix G, HHV-8's lower performance appears to stem from both low coverage and potential sequence similarity to more abundant viral classes, which can introduce confusion during classification. We view this as an important area for future work aimed at improving robustness under such conditions.

Reviewer #3:

Summary:
This paper presents NextVir, a novel framework for classifying oncogenic viruses using genomic foundation models. The study evaluates three advanced models—DNABERT-S, Nucleotide Transformer, and HyenaDNA—to distinguish between different viral genomes, including tumor-associated viruses such as HHV-8 and HPV-16. The models are tested on simulated and real genomic datasets, emphasizing their ability to handle indels, substitutions, and long-context dependencies.Key findings include DNABERT-S outperforming other models in accuracy and robustness due to its advanced fine-tuning with Low-Rank Adaptation (LoRA). Additionally, the paper highlights the computational trade-offs between accuracy and efficiency, with HyenaDNA showcasing speed advantages but struggling with certain viral classes. The robustness of NextVir to mutations and its potential application in metagenomic sequencing are discussed, positioning it as a promising tool for viral research and diagnostics. However, the study identifies limitations such as the reliance on simulated data and emphasizes the need for further validation on clinical samples.
Major comments:

1. While the use of DNABERT-S, Nucleotide Transformer, and HyenaDNA is explained, no comparison is provided against other potential foundational models. Discuss why alternatives like DeepVirFinder were excluded.

We appreciate the suggestion to consider additional models when evaluating the generalizability of the NextVir framework. We would like to clarify that NextVir is designed to operate with genomic foundation models (GFMs) and is therefore not directly comparable to task-specific classifiers such as DeepVirFinder, which is not a general-purpose pre-trained model but rather a model trained specifically for viral sequence detection. In particular, DeepVirFinder outputs a single prediction value instead of a sequence embedding like GFMs. That said, DeepVirFinder is still used as a benchmark in binary classification tasks throughout the manuscript.

The generality of the NextVir framework is demonstrated through its use with three rather diverse GFMs: DNABERT-S, NucleotideTransformer, and HyenaDNA. These models differ substantially in architecture and scale, and can be readily interchanged within the NextVir framework. While we agree that extending this to additional GFMs would provide further insight, current hardware constraints limit our ability to run the even larger models, which require significantly more memory and compute resources than we have access to. In lieu of additional model comparisons, we investigate the impact of other key variables (e.g., sequence length) on NextVir's performance (Appendix I.2), providing further evidence of its flexibility and robustness.

2. The reliance on simulated reads raises concerns about real-world generalizability. Clarify how well the simulated dataset mimics actual tumor samples and address potential biases.

We appreciate the reviewer's concern regarding the use of simulated data and its implications for real-world generalizability. To generate sequencing reads, we used ART, a widely adopted simulator that incorporates empirically derived error profiles to closely approximate the characteristics of real sequencing data. The simulated dataset was not designed to mimic any specific tumor sample, but rather to explore a diverse oncoviral landscape for the purpose of training an effective classifier. Our focus was on ensuring that the model could learn generalizable features of oncoviral sequences, rather than replicating a specific tumor environment.

To assess how well the trained model transfers to real-world data, we include an evaluation in Appendix H where a model trained entirely on simulated reads is applied to real cervical cancer samples. This test provides initial evidence of the practical applicability of the proposed framework.

3. Beyond accuracy, consider reporting F1 score, precision, and recall to provide a more comprehensive evaluation, especially for low-representation classes like HHV-8.

We appreciate the reviewer's recommendation to include additional performance metrics beyond accuracy. In response, we have added Appendix G, which reports class-specific precision, recall, and F1 scores (particularly for the low-representation HHV-8 class) and discusses the inherent challenges of classifying low-coverage viral genomes.

HHV-8 remains a particularly difficult class to distinguish. All models exhibit relatively low per-class F1 and precision scores for HHV-8, reflecting both the limited number of reads and the sequence similarity to more abundant viral classes. Notably, while few reads are incorrectly classified as HHV-8, the class's

small proportion in the overall mixture results in limited opportunities for the model to learn a distinct representation. The additional metrics provide a more nuanced picture of model performance and underscore the importance of future work focused on improving classification under low-coverage and high-similarity conditions.

4. Discuss in more depth why some models are more robust to indels and substitutions. Relate this to tokenization schemes and architectural differences.

Substitutions are relatively localized perturbations so all three tokenization schemes (Byte-Pair Encoding (BPE) in DNABERT-S, non-overlapping k-mers in NucleotideTransformer and 1-mers in HyenaDNA) appear to tolerate the substitutions to a degree. For example, BPE may be resilient to single-base substitutions if the token boundaries remain unchanged, while NucletideTransformer may maintain robustness unless substitutions alter sequence across too many k-mers. On the other hand, indels introduce frame shifts that can disrupt token boundaries. This renders the Nucleotide Transformer particularly sensitive to such mutations, as its entire representation built atop of non-overlapping k-mers could be corrupted. BPE is also more sensitive to indels than to substitutions due to the distortion of token boundaries. HyenaDNA should, in principle, be less sensitive to time shifts but is still affected by them since it architecturally relies on global sequence structure. The architecture of DNABERT-S and NucleotideTransformer, on the other hand, may help mitigate local corruption through smoothing as it attends to token relationships via self-attention.

5. Include comparisons to simpler deep learning models (e.g., CNNs or LSTMs) trained on the same dataset to contextualize the performance gains of NextVir.

We agree with the reviewer that comparing against simpler deep learning models provides valuable context for evaluating the performance gains of NextVir. To this end, our binary classification benchmarks already include models with standard, lower-complexity architectures: DeepVirFinder (CNN-based), Virtifier (LSTM-based), and XVir (transformer-based). These models were trained and evaluated on the same datasets as the binary variants of NextVir, allowing for a direct and fair comparison. Their inclusion helps highlight the performance benefits of incorporating genomic foundation models within the NextVir framework.

6. Clarify how the random and context-supported data splits were constructed and whether they accurately reflect sequencing scenarios in real-world studies.

In the random split, the reads were randomly partitioned into training, validation and test sets without regard to their position along the genome. While this assumes uniform and complete coverage, which is somewhat unrealistic in real-world settings, the random split remains a valid and widely used evaluation strategy for benchmarking that reflects how well a model may perform in research (or best-case clinical) settings. The context-supported data split, on the other hand, aims to emulate non-uniform coverage and localized amplification biases that may occur in sequencing protocols (e.g., PCR-based enrichment). Specifically, after aligning the reads to a reference, we use the first 80% (by position) for training, next 10% for validation, and the final 10% for testing. This ensures there is no overlap in genomic regions between training and testing dataset. The aim of such a setting is to challenge the model to generalize beyond the exact regions it has seen during the training, and to test its predictive capabilities that may otherwise be obscured (i.e., we are trying to make sure the model is not simply memorizing local

sequence motifs). An illustration and further motivation behind context-supported classification is included in Appendix C.

Minor comments:
1. Tables: Include confidence intervals or standard deviations in accuracy results to highlight variability across runs.

Thank you for this suggestion. We have added 95% confidence intervals to Table 2, as calculated through 5 uniquely seeded training runs. Due to the large computational cost of running each experiment an additional 4 times, we've elected to add them to only the primary multi-class results.

2. Inconsistent verb tenses: In the "Results" section, the tense alternates between present and past. Ensure consistency by choosing either present (preferred for describing findings) or past tense.

We thank the reviewer for bringing this to our attention. We have revised the Results section to ensure consistent verb tense throughout, using the present tense as appropriate for describing findings.
