# Supplementary material for: NextVir: Enabling classification of tumor-causing viruses with genomic foundation models
Source: PLoS Comput Biol. 2025 Aug 21;21(8):e1013360. doi: 10.1371/journal.pcbi.1013360 (PMC12396758; doi:10.1371/journal.pcbi.1013360)
Supplement: Attachment — Submitted filename: NextVir_r2revision_response.pdf [file pcbi.1013360.s003.pdf]

## Response to reviews

*Reviewer #1: The manuscript has been comprehensively revised with additional supporting materials. I'm now satisfied with the current version and recommend acceptance as it is.*

*Reviewer #2: We appreciate the authors for revising the manuscript. Compared with the initial version, the manuscript has been significantly enhanced in multiple dimensions, particularly in the detailed elaboration of the NextVir model's performance, which makes the content presentation more coherent and clear. However, upon reviewing the revised manuscript, we identified that some issues remain inadequately addressed. To ensure the high quality of the article, we suggest the authors further refine it accordingly.*

*In the response letter, the authors stated that they had "dedicate the second paragraph of the new Discussion section to comparing the three foundation models used in our work". Nevertheless, the revised content remains at a general level of discussion without providing specific model evaluation metrics or citing authoritative scientific references. We recommend that the authors supplement concrete model evaluation indicators or incorporate relevant authoritative research when revising, thereby strengthening the objectivity and persuasiveness of the comparison.*

*Reviewer #3: The author answered my doubts.*

We thank the reviewers for their time and effort taken to review our work. We are grateful to Reviewer 1 and Reviewer 3 for their approval of the changes made to the manuscript. Moreover, we are grateful for the additional opportunities for improvement pointed out by Reviewer 2, as elaborated below.

To address Reviewer 2’s comment regarding the comparison of foundation models, we have revised the corresponding paragraph in the *Discussion* section to provide a more specific and structured comparison of the genomic foundation models used in this work – DNABERT-S, Nucleotide Transformer, and HyenaDNA. While, to our knowledge, no prior study directly compares **fine-tuned** versions of all three models, we now incorporate findings from a recent benchmark that evaluates their **pretrained** counterparts. Notably, the observations in that study closely align with the trade-offs we report. Accordingly, we have expanded our discussion by referencing this benchmark alongside the original manuscripts for each model, and we now explicitly comment on relevant evaluation metrics such as accuracy, robustness to mutations, and computational efficiency. We hope this expanded comparison directly addresses the reviewer’s concern and offers a clearer perspective on the relative strengths and limitations of each foundation model.
